# The early use of botulinum toxin in post-stroke spasticity: study protocol for a randomised controlled trial

**DOI:** 10.1186/1745-6215-15-12

**Published:** 2014-01-08

**Authors:** Cameron Lindsay, Julie Simpson, Sissi Ispoglou, Steve G Sturman, Anand D Pandyan

**Affiliations:** 1 Physiotherapy Department, Sandwell and West Birmingham NHS Trust and School of Health and Rehabilitation, Keele University, Mackay Building, Keele, Staffordshire ST5 5BG, UK; 2 Pharmacy Department, Sandwell and West Birmingham NHS Trust, City Hospital Birmingham, Dudley Road, Birmingham, West Midlands B18 7QH, UK; 3 Department of Elderly Care, Sandwell and West Birmingham NHS Trust, City Hospital Birmingham, Dudley Road, Birmingham, West Midlands B18 7QH, UK; 4 Department of Neurology, Sandwell and West Birmingham NHS Trust, City Hospital Birmingham, Dudley Road, Birmingham, West Midlands B18 7QH, UK; 5 School of Health and Rehabilitation, Keele University, Mackay Building, Keele, Staffordshire ST5 5BG, UK

**Keywords:** Spasticity, Stroke, OnabotulinumtoxinA, Contractures, Randomised controlled trial, Protocol

## Abstract

**Background:**

Patients surviving stroke but who have significant impairment of function in the affected arm are at more risk of developing pain, stiffness and contractures. The abnormal muscle activity, associated with post-stroke spasticity, is thought to be causally associated with the development of these complications. Treatment of spasticity is currently delayed until a patient develops signs of these complications.

**Methods/Design:**

This protocol is for a phase II study that aims to identify whether using OnabotulinumtoxinA (BoNT-A) in combination with physiotherapy early post stroke when initial abnormal muscle activity is neurophysiologically identified can prevent loss of range at joints and improve functional outcomes.

The trial uses a screening phase to identify which people are appropriate to be included in a double blind randomised placebo-controlled trial. All patients admitted to Sandwell and West Birmingham NHS Trust Hospitals with a diagnosis of stroke will be screened to identify functional activity in the arm. Those who have no function will be appropriate for further screening. Patients who are screened and have abnormal muscle activity identified on EMG will be given electrical stimulation to forearm extensors for 3 months and randomised to have either injections of BoNT-A or normal saline. The primary outcome measure is the action research arm test - a measure of arm function. Further measures include spasticity, stiffness, muscle strength and fatigue as well as measures of quality of life, participation and caregiver strain.

**Trial registrations:**

ISRCTN57435427, EudraCT2010-021257-39, NCT01882556

## Background

Stroke is a common cause of disability and is a significant contributor to loss of independence [1]. Those who have not recovered some level of useful function in the acute stages (within 4 to 6 weeks of the stroke) are at more risk of developing long-term musculoskeletal complications such as contractures and pain [2-4]. It is generally believed that the lack of functional recovery and these secondary complications result from the development of spasticity, that is, intermittent or sustained involuntary activation of muscles [5] that normally precedes the development of these secondary complications.

The management of spasticity is complex and normally involves multiple professions [6]. Rehabilitation therapies are generally eclectic and are aimed at preventing the secondary complications and reducing aggravating factors rather than the abnormal muscle activity itself [6]. Treatment of spasticity, in current practice, is delayed until secondary complications are established. It is possible that deterioration in the musculoskeletal impairments before the initiation of spasticity treatment may be detrimental to the patient as these musculoskeletal changes may have become too chronic and impossible to fully eradicate [7].

This study will evaluate the benefit of early treatment with OnabotulinumtoxinA (BoNT-A) when added to standard care therapies, in patients suffering from post-stroke upper limb spasticity, which is identified in its early stages by clinical and neurophysiological markers.

The objective of this study is to investigate whether the combination of decreasing the abnormal muscle activity of forearm flexors results in greater functional recovery than increasing the extensor activity alone.

## Methods and design

### Objectives

#### Primary objective

To evaluate the clinical effects of BoNT-A and physiotherapy when compared with placebo and physiotherapy, in patients with focal spasticity post stroke, identified on clinical and neurophysiological grounds, in facilitating the recovery of arm function (measured using the Action Research Arm Test (ARAT)).

#### Secondary objectives

1. To evaluate the effectiveness of BoNT-A and physiotherapy when compared with placebo and physiotherapy in patients with focal spasticity, post stroke, identified on clinical and neurophysiological grounds.

2. To reduce focal spasticity in the arm as measured by surface EMG response of the wrist and elbow flexors to an externally imposed perturbation.

3. To improve strength and fatigue as measured by maximum isometric strength and the rate of force production in the wrist and elbow joints.

4. To reduce stiffness and increase passive range of movement by measuring the range of movement and force required to produce the movement with a custom built device.

5. To reduce post-stroke pain measured using a visual analogue scale.

6. To improve quality of life (using the EuroQol Group EQ5D) and assess care giver burden (using the Care Giver Burden Scale).

7. To reduce the need for additional oral anti-spasmodic drugs or additional botulinum treatment during the course of rehabilitation.

8. To reduce long-term costs (quantified using resource utilisation diaries) and identify discharge destination.

### Study design

This study combines a screening phase to identify appropriate subjects to enter a phase II, double blind, randomised, placebo-controlled trial. Final follow-up will be at 6 months following stroke.

### Ethics

This study was approved by North West - Greater Manchester South Ethics Committee Reference number 10/H1003/111.

### Recruitment

All patients admitted to the Trust with a diagnosis of stroke will be eligible to participate. Patients will be identified as potential candidates for recruitment during the initial (standard practice) assessment by the Stroke Physician or Physiotherapist. Patients and relatives will then be approached by the Trial Clinician. A decision will be made on the patient’s capacity to consent 24 hours after initially being approached by the Trial Clinician. In cases where the patient lacks capacity a legal representative will provide consent.

### Inclusion criteria to screening phase

• Over 18 years of age.

• Patients admitted to hospital with a diagnosis of stroke (between days 1 and 42) due to a primary cerebral haemorrhage/infarction or subarachnoid haemorrhage producing an upper motor syndrome affecting one body side which results in a hemiplegia.

• Capable of providing informed consent directly or indirectly, or, consent obtainable from next of kin or legal representative.

• No useful arm function (Defined as less than or equal to 2 on the grasp subsection of the ARAT) at onset of spasticity.

### Eligibility for randomisation to treatment phase of study

To be eligible for the treatment phase of the study patients will have to meet the following additional criteria.

• Evidence of upper limb spasticity demonstrated by a muscle response during a passive stretch of a relaxed muscle.

### Exclusion criteria

• Significant musculoskeletal conditions that affected upper limb function prior to the stroke (for example, pre-existing contractures).

• Unconscious or moribund during the screening period.

• Recovery of useful arm function (a score of 3 or more in the grasp section of the ARAT) prior to injections.

• Patients with contraindications to electrical stimulation including active implants (for example, cardiac assist devices), metal implants at site of stimulation, scar tissue/cancerous tissue at site of stimulation, uncontrolled epilepsy, deep vein thrombosis in limb/muscle being stimulated and pregnancy (or planned pregnancy). These will be dealt with on a case-by-case basis.

• Previous upper motor neurone syndrome or hypertonicity due to multiple sclerosis, spinal cord injury or other neurological disorder.

• Patients with a known hypersensitivity to any BoNT-A or to any of the excipients of BoNT-A (for example, human serum albumin).

• Patients with myasthenia gravis or Eaton Lambert syndrome or other neuromuscular junction or myopathic disorder.

• Patients with infection at the proposed injection site(s).

• Patients who are pregnant or may become pregnant at the time of the proposed injections and for the duration of the study.

• Current treatment with any anti-spasticity agent or previous injection with BoNT-A.

### Screening phase

From the date of consent, Patients who are enrolled in the study will be monitored for a period of 6 weeks from stroke onset by an independent assessor (the study therapist). Monitoring will normally be carried out every other day excluding weekends. The frequency will be increased to daily if clinically indicated. For monitoring purposes the therapist will conduct two simple bedside tests: (1) the grasp subsection of the ARAT; and (2) the surface EMG response of the wrist and elbow flexors to a passive externally imposed stretch. (Non-invasive EMG electrodes will be placed on the elbow flexors and wrist flexors.) The joint limb will be moved from full flexion to extension a maximum of six times from a position of rest. Presence of spasticity will be a velocity related increase in muscle activity. Patient positioning will be documented as sitting or half lying.

### Study groups

During the screening phase, patients will be categorised into one of the three following groups depending on their presentation.

Group one: the patient recovers function. On recovery of function a full set of baseline measures will be taken and follow-up at 3 and 6 months will be planned.

Group two: the patient develops no abnormal muscle activity in the first 42 days following the stroke. At day 42 a full set of baseline measures will be taken and follow-up at 3 and 6 months will be planned.

Group three: the patient develops abnormal muscle activity and no functional recovery has occurred. The patient will have a full set of baseline measures taken and then be randomised.

### Randomisation phase

#### Interventions

All eligible patients were randomised to receive I.M. injections of BoNT-A or 0.9% sodium chloride solution to 6 muscles of the affected arm in predetermined doses. Muscles that will be injected are flexor digitorum superficialis, flexor digitorum profundus, flexor carpi ulnaris, flexor carpi radialis, biceps and brachialis. The volume to inject will be calculated on the number of units per mL that the injection solution would contain if it contained BoNT-A. Table 1 shows the OnabotulinumtoxinA units to be administered to each muscle. In patients with a substantial lack or excess of muscle bulk or where there is excessive muscle activity leading to clonus larger or smaller doses will be administered (as shown in Table 1) at the discretion of the research clinician.

**Table 1:** OnabotulinumtoxinA units for muscles to be injected

**Muscle**	**Units (−25%)**	**Main dose units**	**Units (+25%)**
Biceps	30	40	50
Brachialis	30	40	50
Flexor digitorum superficialis	20	25	30
Flexor digitorum profundus	20	25	30
Flexor carpi ulnaris	10	15	20
Flexor carpi radialis	10	15	20

### Randomisation method

Randomisation was by computer-generated random permuted blocks in a pseudorandom sequence.

### Blinding

The research clinician will complete the prescription form and, following assessment of the patient by a Consultant, the prescription will be signed by the Consultant. The prescription will then be taken to pharmacy where either two vials of BoNT-A and one 5 mL ampoule of 0.9% sodium dhloride solution (Treatment group) or only the one 5 mL ampoule of 0.9% sodium chloride solution (Placebo group), depending on the randomisation, will be dispensed.

The dispensed drug will be taken to the ward in a sealed opaque bag where an independent clinician will fill the syringes with either the reconstituted toxin in solution or solution alone depending on the randomisation. A second trained clinician will be in attendance to confirm the syringes were filled with dispensed product. Separate sharps bins will be used for preparation/reconstitution and injecting. This will ensure that the patient and injector will remain blinded to treatment.

Localisation of the involved muscles will be determined by electrical stimulation techniques. Where localisation of the muscles for injection is not clear using EMG then ultrasound should be employed to guide the injection procedure and check accuracy of placement of the needle in the specified muscles.

### Outcome measurements

Arm function was measured using the ARAT [8]. The measure will be carried out by a single researcher at baseline, 3 months post injection and 6 months post stroke using the standardised approach advocated by Yozbatriran et al. [9].

### Measures of spasticity and contractures

Spasticity in the wrist and elbow flexors will be quantified by measuring the muscle activity and stiffness, during an externally imposed stretch of a relaxed muscle. Two stretch velocities will be used. In addition to quantifying spasticity (from the EMG data) and contractures (stiffness) from the data collected during this procedure it will be possible to quantify passive range of movement and the data required to provide a Tardieu Score related to spasticity. A flexible electrogoniometer will be placed on the lateral border across the wrist or elbow - this allows for non-invasive measurement of joint range of movement.

These measurements were taken using the following standardised protocol. Surface EMG electrodes will be placed in accordance with European recommended sites for the biceps and long head of triceps [10].

The electrodes will be placed on the forearm flexor and extensor muscles using bony landmarks to identify location as follows:

Forearm flexor muscles - In a line between the medial epicondyle of the humerus and medial border of the biceps tendon - from the mid-point of this line the electrodes will be attached approximately one-third of the length of the forearm. With the arm in full pronation the electrode is placed one-third of the distance between the lateral epicondyle of the humerus and the radial styloid.

The therapist will move the joint from full flexion to full extension, using two manually controlled velocities, a maximum of six times. The data will be collected using a DataLog (Biometrics Ltd.). Following transfer to a computer, the data will be interrogated to produce measurements of spasticity and joint stiffness. Patient position will be uniform at all measurements. This measurement protocol has been used in previous trials [11,12].

These measures will be taken at baseline, 2, 4 and 6 weeks and 3 months post injections and finally at 6 months post stroke.

Pain will be measured using a visual analogue scale at baseline, 3 months and 6 months.

Muscle function will be quantified by measuring isometric strength at mid-range of movement using a dynamometer. A grip dynamometer will measure grip strength. (Fatigue will be derived from the above measurement of strength by estimating the time taken for the force generation to drop to 70% of the maximum isometric strength measurement). These measures will be taken at baseline, 3 months and 6 months.

The Barthel index will be measured at baseline, 3 months and 6 months [13]. While it was originally devised to assess the individual’s capacity to carry out physical tasks, it has subsequently become used as a measure of activity.

Measure of quality of life using the EuroQuol EQ5D will be taken at 3 and 6 months. The EuroQuol EQ-5D-5 L is a health-related measure of quality of life measuring five dimensions: mobility, self-care, usual activities, pain/discomfort and anxiety/depression. While the similar but older version (5D-3 L) with just three levels of problem has been used extensively in stroke, the newer version with five levels has been used less so.

A measure of care giver burden will be taken at 6 months using the Care Giver Strain Index (CGSI) as will the modified Rankin scale. The CGSI was first developed by Robinson (1983) and has been used widely with stroke survivors’ families and carers [14].

### Planned statistical analysis

The study team will assess the data collected for all patients at a review meeting to determine the suitability of each patient in the analysis populations prior to breaking the blind for the whole group. AE reporting will be based on the intention to treat population.

### All-available-patient (AAP) population

All patients who have consented for the study will be used for patient accountability and listings and will include patients who are randomised but who do not receive their injection of study medication.

Intent-to-treat (ITT) population: All patients who have been randomised and received their injection of study medication at baseline will be included in an ITT population (missing values will be dealt with using standard procedure in current use). All patients included in the ITT population, who participate in the study, without major protocol violation.

The study team with guidance from a medical statistician and health economist will assess the data collected for all patients at a review meeting to determine the suitability of each patient in the analysis populations prior to breaking the blind for the whole group. AE reporting will be based on the ITT population.

Demographic and background data will be summarised using appropriate summary statistics. Effects of treatment will be estimated using effect size calculations (difference between groups divided by the pooled standard deviation) and 95% confidence interval of the effect size for all continuous variables and odds ratio with 95% confidence intervals will be used for categorical variables (for example, pain).

### Sample size calculations

Sample size calculations have been identified using data from a pilot study [12]. With an effect size of 0.5 and at 80% power (0.05) significance level 126 patients will be required. With an effect size of 0.6 and at 80% power (0.05) significance level 88 patients will be required. Allowing for an attrition of 20% the maximum number of participants we will need is 150 and the minimum is 88.

All available patients who have consented for the study will be used for patient accountability and listings and will include patients who are randomised but who do not receive their injection of study medication.

All patients who have been randomised and received their injection of study medication at baseline will be included in an ITT population (missing values will be dealt with using standard procedure in current use).

### Dissemination plan

The results of the trial, regardless of outcome, will be disseminated through the traditional routes of scientific peer-reviewed publications, international and national specialist conferences, and the Stroke Research Network. CL will be responsible for initially drafting these manuscripts and professional writers will not be used for any of the publications. Authorship will be based on the criteria defined by the International Committee of Medical Journal Editors [15]. We aim to be able to compose the final results paper within 4 months of the unblinding procedure occurring. Subjects who have been involved in the trial will be given the option of having a summary of the results sent to them.

## Trial status

Recruitment began in February 2012 and is due to end in December 2013.

## Abbreviations

ARAT: Action research arm test; BoNT-A: OnabotulinumtoxinA; CGSI: Care giver strain index; EMG: Electromyogram.

## Competing interests

Allergan Ltd. is providing the botulinum toxin type A free of charge. All elements of the study are being undertaken independently of Allergan Ltd. CL, ADP and SGS have previously received honorarium from both Allergan Ltd. and Ipsen Ltd. for training and teaching. ADP has also received honorarium from Merz Ltd for teaching. JS and SI have no competing interests.

## Authors’ contributions

CL conceived the study, acquired the funding, coordinated and participated in the design of the study and drafted the manuscript. JS participated in the design and implementation of the study and provided critical revision to this manuscript. SI participated in the implementation of the study and provided critical revision to this manuscript. SS participated in the design of the study. ADP conceived the study, acquired the funding, and participated in the design of the study and provided critical revision to this manuscript. All authors have approved the final manuscript.
